# Spray-dried plasma protects against rotavirus-induced gastroenteritis via regulating macrophage and T cells divergence in weanling pigs

**DOI:** 10.3389/fvets.2024.1467108

**Published:** 2024-10-16

**Authors:** Hui Yan, Biqiong Dong, Xipeng Li, Jun He, Bing Yu, Xiangbing Mao, Jie Yu, Yuheng Luo, Junqiu Luo, Aimin Wu, Junning Pu, Quyuan Wang, Huifen Wang, Joe Crenshaw, Yanbin Shen, Daiwen Chen

**Affiliations:** ^1^Key Laboratories for Animal Disease-Resistance Nutrition of China Ministry of Education, China Ministry of Agriculture and Rural Affairs and Sichuan Province, Animal Nutrition Institute, Sichuan Agricultural University, Chengdu, China; ^2^R&D Department, APC LLC, Ankeny, IA, United States

**Keywords:** spray-dried plasma, macrophage, T cell, intestinal health, viral gastroenteritis, weanling pigs

## Abstract

Infectious gastroenteritis is the major cause for diarrhea in piglets. The protection of spray-dried plasma (SDP) on viral gastroenteritis during the progression of rotavirus (RV) infection remain unclear. In this study, 64 weanling piglets were randomly assigned to control diets (*n* = 40) and SDP diets (*n* = 24) for 14 days, and then pigs were challenged with RV on day 15. Pigs were sacrificed on day 14 (normal condition), day 18 (manifestation stage), and day 21 (convalescent stage) of the trial. Prior to RV infection, SDP increased ADG, M1 macrophages and CD4^+^ T cells in different organs without increasing proinflammatory cytokines, indicating a more robust immunity with less inflammation. During the manifestation of infection, SDP enhanced mucosal immunity by increasing M1 macrophages, M1/M2 ratio and cytokines in mucosa and increasing intraepithelial CD8^+^ T cells for RV clearance. During the convalescence, SDP promoted M2 macrophage polarization and reduced pro-inflammatory cytokines to facilitate intestinal repair and prevent prolonged inflammation. Collectively, SDP enhanced mucosal immunity to promote viral clearance and maintained immune homeostasis to prevent long-lasting inflammation as a therapeutically approach for infectious gastroenteritis.

## Introduction

Infants and young animals are susceptible to various gastrointestinal disorders due to underdeveloped intestinal barrier and immune system, leading to nutrient malabsorption, diarrhea, and even death (1–3). Infectious gastroenteritis, caused by rotavirus, norovirus, *Escherichia coli* and other pathogens, is the most common gastrointestinal disorder predominately in young animals (4). Rotavirus primarily infects enterocytes and impairs intestinal barrier function, causing acute gastroenteritis and dehydration (5–7). Rotavirus gastroenteritis remains the major viral cause for acute diarrhea in both children and young animals globally, causing severe health issues and tremendous economic losses in hospitalizations and livestock production (8, 9).

Spray-dried plasma (SDP) is primarily used in weanling pig diets, and has been shown to improve growth performance and intestinal integrity (10, 11). Recent studies have shown that SDP accelerates the clearance of porcine rotavirus (RV) and porcine epidemic diarrhea virus (PEDV), both of which are major causes of intestinal diseases in pigs (12, 13). Administration of SDP was reported to impede the infectivity of the porcine reproductive and respiratory syndrome virus (PRRSV) and African swine fever virus (ASFV) in pigs (14, 15). Furthermore, studies have shown that spray-dried plasma (SDP) alleviated intestinal inflammation and promote antibody production upon PEDV infection (16). Despite these findings, the mechanisms by which SDP modulates the immune response involved in resisting pathogen infections, remain poorly understood. Animals rely on the immune system against pathogen infection. Research indicated that macrophages are responsible for clearing pathogens and damaged cells, as well as supporting tissue repair. Dysfunction in macrophage activity, particularly an imbalance in the M1/M2 ratio, is associated with intestinal inflammation and tissue damage (17). T cells are involved in pathogen recognition and regulatory functions within the intestine. Imbalances in T cell function can lead to excessive inflammation and non-specific antigen responses (18). However, the precise immunological modulation by which SDP regulates immune cell populations, particularly at different stages of viral infection, is yet to be identified.

In this study, we established a swine model of rotavirus gastroenteritis by challenging weanling pigs with porcine rotavirus (RV) and investigated the disease progression and immune responses at different infection stages. We further examined the role of SDP in the protection of intestinal barrier integrity via the regulation of monocyte-macrophage systems and lymphocyte populations during RV infection. Our study seeks to explore the progression of gastroenteritis and immune responses in weanling pigs upon RV infection and provide a nutritional solution to alleviate the intestinal damage for young animals.

## Materials and methods

### Animal ethics statement

All animal studies and protocols were approved by the Sichuan Agricultural University Animal Care and Use Committee (20230391) for the compliance with ethics requirements.

### Animals and experimental design

All experiments were conducted at the research facility at Sichuan Agricultural University in China. Experimental piglets were randomly sampled for testing of African swine fever, *Escherichia coli*, rotavirus, classical swine fever, and other pathogens prior to transport to the research farm. The enclosure was thoroughly disinfected.

A total of 64 healthy weanling pigs [(Duroc × Landrace × Yorkshire, barrows) weaned at 18-day of age with 3 days of acclimation and an initial body weight of 6.0 ± 0.5 kg] was randomly assigned to 2 experimental diets, including the control diet (basal diet with 6% soybean protein isolate, *n* = 40) and the SDP diet (basal diet with 6% SDP, *n* = 24) for 14 days. On the day 15, pigs were challenged with RV for 3 to 6 days, respectively.

Animals were sacrificed at 3 different stages as following: On day 14, prior to RV challenge, 8 pigs were randomly selected from each treatment for sampling as the normal stage (*n* = 8). The rest of pigs were further divided into 3 sub-groups with16 animals in each gourp for RV challenge: (1) control diet without RV infection; (2) control diet with RV infection; (3) SDP diet with RV infection. On day 3 (manifestation stage, *n* = 8) and day 6 (convalescent stage, *n* = 8) of RV challenge, 8 pigs from each treatment were sacrificed for sampling, respectively.

The pigs were individually housed in a nursery crate in a controlled environment and were fed *ad libitum* with free access to water. All diets were formulated with equal amounts of crude protein and energy and met the nutrient requirements (NRC 2012). The ingredients and chemical composition are shown in Supplementary Table S1.

### Porcine rotavirus preparation and challenge

The RV used in this study was a tissue culture-adapted Ohio State University (OSU) strain (ATCC#VR-893) propagated in MA104 cells. The RV used in the experiment had a viral titer of 10^6^ TCID_50_/mL. On the day 15, all pigs were infused with 50 mL of sterile NaHCO3 solution (100 mmol/L) to neutralize the pH of the gastric juice. After 1 h, 16 piglets randomly selected from the control group or the SDP group received 4 mL (10^6^ TCID_50_/mL) of RV solution by oral gavage, and the remaining piglets in the control group received the same dose of saline. The infusion concentration was based on the previous publication, and the preliminary experiment was also conducted to ensure that this concentration caused diarrhea in pigs in approximately 3 days (19).

During the experiment, the daily feed intake (FI) was recorded, and their body weight (BW) was measured at the beginning and end of each week to calculate the average daily gain (ADG), average daily feed intake (ADFI), and feed-to-gain ratio (F/G). Fresh faeces were scored according to the following criteria: 0 for normal (firm faeces), 1 for soft consistency (soft and moulded faeces), 2 for liquid faeces, and 3 for severe diarrhea (watery faeces). Diarrhoea was recorded if the faeces score was greater than 2.

### Sample collection

During each study stage normal stage: day 14 of the trial; manifestation stage: day 3 after RV challenge (day 18 of trial); and convalescent stage: day 6 after RV challenge (day 21 of the trial), eight piglets per treatment group were randomly chosen for sampling. The manifestation and convalescent stages were defined based on the diarrhea progression in the challenged piglets. Diarrhea peaked on the 3th day post-challenge, and by the 6th day, nearly all piglets had recovered. Accordingly, samples were collected at these two time points, which were designated as distinct stages.

Blood samples were extracted from the anterior vena cava and collected in vacuum tubes. The piglets were then anaesthetized through intravenous injection of pentobarbital sodium and euthanized. Samples of the jejunum and ileum were gathered and immersed in a 4% paraformaldehyde solution for the purpose of intestinal morphology evaluation. Furthermore, specimens of the intestinal contents and mucosa were collected and preserved at −80°C for subsequent analysis.

### Intestinal morphology

Intestinal specimens fixed in 4% paraformaldehyde were dehydrated in low- to high-concentration ethanol, embedded in paraffin, sectioned at 5 μm thickness, and stained with haematoxylin and eosin (H&E). At least 10 structurally complete villi and crypts were selected for each section, and the total villus length and crypt depth were measured using a digital microscope (Image Pro Plus 6.0) at a magnification of 40× to calculate the average villus height, crypt depth, and villus height-to-crypt depth ratio (V:C).

### Total RNA extraction and real-time quantitative PCR

Total RNA was extracted from jejunum mucosa samples using the Trizol reagent according to manufacturer’s instructions (Takara Bio, 9109). The extracted total RNA was reverse transcripted using PrimeScript^™^ RT reagent and gDNA Eraser (Takara Bio, RR047A). The qPCR reaction utilized the nucleic acid dye SYBR/EVA Green (Takara Biotech). The double ΔCt method was used to determine the relative mRNA expression against internal reference. The primers sequences used in this study were shown in Supplementary Table S2.

### Cytokines and immunoglobulin measurement in serum and ileal mucosa

The levels of cytokines, interleukin-6 (IL-6), IL-2, TGF-β, IL-1β, IL-4, IL-10, tumor necrosis factor-α (TNF-α), and interferon-γ (IFN-γ) were measured in serum and ileal mucosa using commercially available porcine ELISA kits (Jiangsu Jingmei Biotechnology Co., Ltd., Yancheng, China). Serum D-lactate diamine (DAO), immunoglobulin (Ig) A, IgG, and IgM concentrations, and the ileal secretory IgA (sIgA) level were determined using commercially available porcine ELISA kits (Jiangsu Jingmei Biotechnology Co., Ltd., Yancheng, China). Serum RV antibody levels were measured using ELISA kits (omnim.Abs Co., Ltd., United States). All tests were performed according to the manufacturer’s instructions.

### Immune cell subtype identification

Single-cell suspensions were prepared from intraepithelial cells and Peyer’s patch (PP) in jejunum, mesenteric lymph nodes (MLN), and spleen by processing fresh tissues with rinsing, collagenase digestion and filtration according to the previous publication (20, 21). Fluorescence-activated cell sorting (FACS) was conducted in Flow cytometer and analyzed in FlowJo software (FlowJo LLC) to determine the macrophage and lymphocyte populations. Cell staining and gating strategies were detailed as follows:

Macrophage sorting: The surface staining process with antibodies to FITC CD45, APC/Cyanine7 CD86, and PE/Cyanine7 CD206 was conducted per the manufacturer’s instructions, except that in the present experiment, immune cells were initially labeled with CD45, and CD86 (M1) and CD206 (M2) were labeled subsequently, based on CD45.

Lymphocyte sorting: The methodology used for surface staining with antibodies to Alexa Fluor^®^647 CD45, PE-Cy^™^7 CD3, PerCP-Cy^™^5.5 CD4a, and PE CD8 adhered to the manufacturer’s instructions. CD45 was initially used to mark immune cells. Subsequently, CD3 was used to mark lymphocytes based on CD45, and CD8 (cytotoxic T lymphocytes) and CD4 (helper T lymphocytes) were marked based on CD3.

### Statistics analysis

Statistical analysis was conducted using GraphPad Prism 9.0 software. Significance was assessed using student’s *t*-test and ANOVA with appropriate post-hoc multiple comparisons. For unbalanced data, Welch’s *t*-test was applied. Non-parametric tests were used for data that did not follow a normal distribution. The data are presented as mean ± SEM. ^*^p < 0.05, ^**^p < 0.01 and ^***^p < 0.001.

## Results

### Spray-dried bovine plasma increases growth performance of weanling pigs

The growth performance of the weanling pigs was detailed in Table 1. Prior to the RV challenge, pigs fed the diet with SDP exhibited a tendency to increase body weight (BW) compared with the control diet, even within a short period of feeding. In the 1st week of the experiment, the feed conversion ratio (F:G) of piglets in SDP group was significantly lower than that of control piglets (*p* = 0.044). In the 2nd week, piglets in SDP group showed a significantly higher average daily gain (ADG) than control group (*p* = 0.030). Under normal conditions, the weanling pigs fed the SDP diet exhibited a better growth performance than control piglets.

**Table 1:** Spray drying bovine plasma improves growth performance of weaned piglets under normal conditions.

Items	Normal condition		*p*-value
	CON	SDP	
BW, kg			
0 day	6.45 ± 0.05	6.48 ± 0.06	0.713
7 days	7.54 ± 0.13	7.73 ± 0.15	0.341
14 days	10.28 ± 0.23	10.88 ± 0.26	0.087
Diarrheal rate, %	12.33 ± 2.5	9.44 ± 2.69	0.455
1–7 days			
ADG, g	156.99 ± 15.02	175.65 ± 19.18	0.448
ADFI, g	172.98 ± 11.16	166.87 ± 13.88	0.733
F/G	1.12 ± 0.06	0.97 ± 0.04	0.044
8–14 days			
ADG, g	391.35 ± 16.96	450.00 ± 19.91	0.030
ADFI, g	592.44 ± 21.46	625.61 ± 30.35	0.377
F/G	1.53 ± 0.06	1.47 ± 0.09	0.600
1–14 days			
ADG, g	274.17 ± 14.46	312.82 ± 16.84	0.088
ADFI, g	387.75 ± 13.61	396.24 ± 19.62	0.724
F/G	1.40 ± 0.05	1.30 ± 0.04	0.140

ADFI, average daily food intake; ADG, average daily gain; F/G, average daily food intake/average daily gain. Data are presented as mean ± SEM.

During the manifestation phase, RV-challenged piglets exhibited a significant decline in ADG (*p* = 0.004) and feed intake (FI) (*p* = 0.016, Table 2). In RV-infected pigs, SDP increased ADG by 11.6% and decreased F:G by 0.2 points. During the convalescent period, SDP further promoted the recovery of pigs as indicated by the increased ADG and ADFI compared to RV-infected pigs, and the F: G in SDP-treated pigs was similar to the F:G of normal pigs. As a result, SDP exhibited the alleviation on the RV induced reduction of growth performance.

**Table 2:** Spray drying bovine plasma improves growth performance of weaned piglets post rotavirus challenge.

Items	CON	CON-RV+	SDP-RV+	*p*_RV_ ^c^	*p*_SDP_ ^d^
BW, kg					
14 days	10.57 ± 0.31	10.26 ± 0.37	10.69 ± 0.30	0.529	0.366
18 days^a^	12.21 ± 0.36	11.39 ± 0.46	11.80 ± 0.43	0.175	0.522
21 days^b^	13.78 ± 0.6	12.52 ± 0.94	13.87 ± 0.52	0.284	0.238
Diarrheal rate, %					
15–18 days	0	39.58 ± 10.19	33.33 ± 9.13	0.001	0.651
15–21 days	1.04 ± 1.04	39.58 ± 10.08	37.50 ± 7.83	0.001	0.871
ADG, g					
15–18 days	410.58 ± 22.77	283.30 ± 43.15	316.02 ± 34.84	0.004	0.394
19–21 days	444.29 ± 29.81	339.11 ± 58.52	364.11 ± 48.63	0.139	0.748
ADFI, g					
15–18 days	575.37 ± 41.24	401.45 ± 37.88	448.26 ± 38.64	0.016	0.560
19–21 days	588.55 ± 58.46	375.59 ± 50.6	429.09 ± 54.74	0.016	0.485
F/G					
15–18 days	1.43 ± 0.11	1.84 ± 0.38	1.61 ± 0.18	0.315	0.592
19–21 days	1.37 ± 0.19	1.76 ± 0.63	1.39 ± 0.27	0.576	0.610

Data are presented as mean ± SEM.

a 15–18 days: This period represents the manifestation period of the trial process.

b 19–21 days: This period represents the convalescent period of the trial process.

c *pRV: This represents the p-value of CON and CON-RV+.*.

d *p*_SDP_: This represents the *p*-value of CON-RV+ and SDP-RV+.

### Spray-dried bovine plasma improves intestinal health of weanling pigs

**Figure 1:**
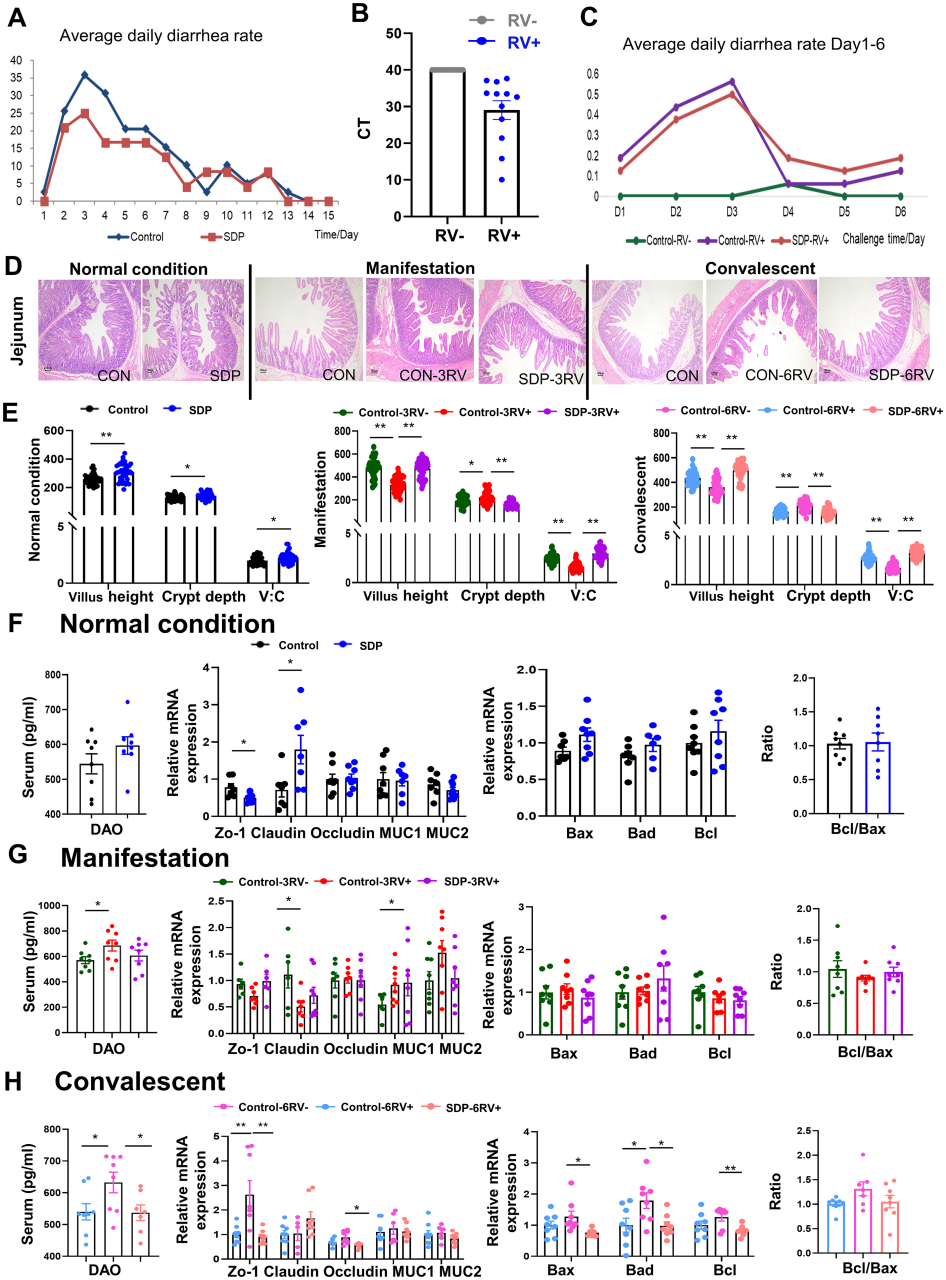
Spray-dried bovine plasma improves intestinal health of weanling pigs. **(A)** Diarrhea rate in control (*n* = 40) and SDP (*n* = 24) groups under normal conditions. **(B)** Expression of RV virus-related proteins in faces of infected and uninfected piglets after RV challenge. **(C)** Diarrhea rates in different treatment groups 1 to 6 days after RV challenge (*n* = 8). **(D)** HE staining of jejunal at different stages Scale bar = 100 μm. **(E)** Under normal condition, manifestation stage and convalescent period, villus length, Crypt depth and villus/crypt ratio of jejunal in different treatment groups were measured. Under normal condition **(F)**, manifestation stage **(G)** and convalescent stage **(H)**, DAO levels in serum and expression of intestinal tight junctions and transporters and apoptosis-related genes in jejunal mucosa in different treatment groups Data are expressed as mean ± SEM (*n* = 8).

Prior to the RV challenge, all weanling pigs suffered from diarrhea in the 1st week, due to weaning stress and environmental changes, but SDP in diet alleviated the diarrhea (Figure 1A). All pigs were RV negative prior to RV challenge; upon RV oral gavage, the RV was detected in anus swab samples and pigs started diarrhea (Figures 1B,C), indicating the successful establishment of the porcine RV infection model.

The intestinal morphology of jejunum was investigated in the section of jejunum by using H&E staining (Figure 1D). Under normal conditions, SDP supplementation significantly increased villus height (*p* < 0.0001) and V:C ratio (*p* = 0.012), as well as decreased crypt depth (*p* = 0.030) in jejunum of weanling pigs (Figures 1D,E). The expression of tight junction and apoptosis related genes were investigated, and *claudin* expression was significantly increased by SDP supplementation (*p* = 0.026, Figure 1F).

Rotavirus infection significantly impaired the structure and integrity of jejunum at manifestation and convalescent stages, as shown by the significant decreases in villus height (*p* < 0.0001) and V:C ratio (*p* < 0.0001) (Figures 1D,E), and the increase in crypt depth (*p* = 0.032) and serum DAO (*p* = 0.042) levels (Figures 1G,H). Notably, SDP in the diet exhibited the protection against RV-induced intestinal damage because SDP significantly increased villus height (*p* < 0.0001) and V:C ratio (*p* < 0.0001) (Figures 1D,E), and decreased crypt depth (*p* < 0.0001) and serum DAO (*p* = 0.039) in RV-infected pigs (Figures 1G,H). RV infection significantly decreased tight junction *ZO-1* (*p* = 0.059) and *claudin-1* (*p* = 0.036) expressions, and increased *MUC1* (*p* = 0.041) expression during the manifestation stage (Figure 1G), and gradually increased *ZO-1* (*p* = 0.015) expressions during the convalescent stage (Figure 1H). RV infection induced expressions of apoptosis related genes Bax, Bad, Bcl during the convalescent stage, indicating the induction of intestinal apoptosis (Figure 1H). Similar to the protection of SDP on intestinal health, SDP in the diet prevented the RV-induced changes in tight junction expressions and intestinal apoptosis at different stages of RV infection (Figures 1G,H). As a result, SDP protected intestinal integrity upon RV infection and maintained intestinal health similar to non-infected pigs.

### Spray-dried bovine plasma modulates production of cytokines in weanling pigs

**Figure 2:**
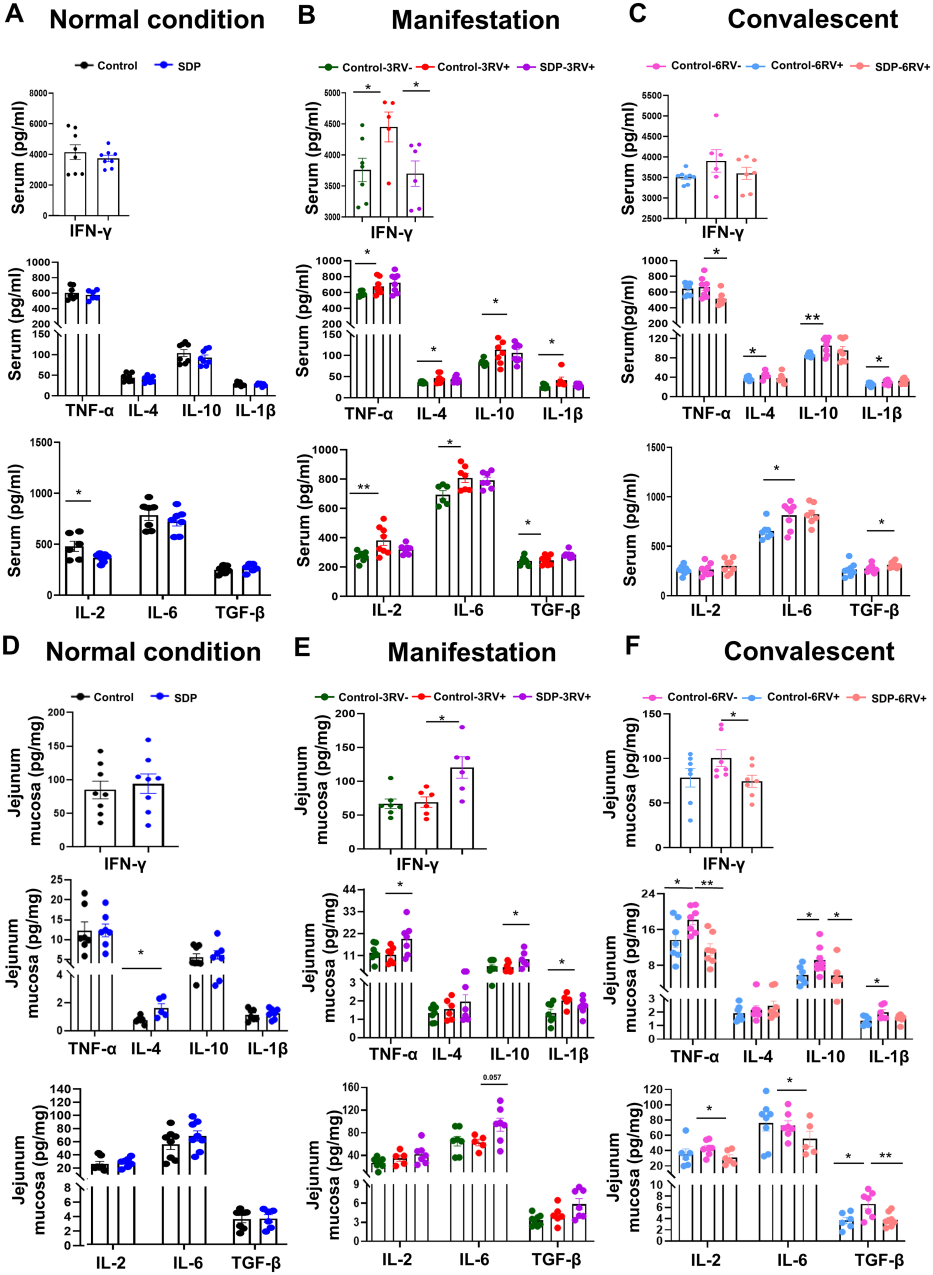
Spray-dried bovine plasma modulates production of cytokines in weanling pigs. Changes in serum levels of inflammatory cytokines in different treatment groups at different stages. [Normal condition stage **(A)**, manifestation stage **(B)** and convalescent stage **(C)**]. Data are expressed as mean ± SEM (*n* = 8). Changes in ileal mucosa levels of inflammatory cytokines in different treatment groups at different stages. [Normal condition stage **(D)**, manifestation stage **(E)** and convalescent stage **(F)**]. Data are expressed as mean ± SEM (*n* = 8).

Innate immunity acts as the first line of defense for pathogen recognition and clearance, immune activation, and tissue repair. Under normal conditions, SDP in the diet significantly decreased serum IL-2 (*p* = 0.042,) and increased jejunal mucosal IL-4 (*p* = 0.019), suggesting an anti-inflammatory potential, but other cytokines levels in serum and mucosa were only slightly influenced (Figures 2A,D).

RV infection significantly increased serum levels of pro-inflammatory cytokines TNF-α (*p* = 0.046), IFN-γ (*p* = 0.045), IL-6 (*p* = 0.020), IL-1β (*p* = 0.047) and IL-2 (*p* = 0.008), and anti-inflammatory cytokines IL-4 (*p* = 0.023) and IL-10 (*p* = 0.024) levels during the manifestation stage, and maintained the high serum levels of IL-1β (*p* = 0.038), IL-6 (*p* = 0.018), IL-4 (*p* = 0.030) and IL-10 (*p* = 0.008) during the convalescent stage (Figures 2B,C), indicating the continuous activation of systemic inflammation. Notably, SDP-treated pigs showed the lower levels of IFN-γ (*p* = 0.039) during the manifestation stage, and the lower TNF-α (*p* = 0.019), higher TGFβ (*p* = 0.046) levels during the convalescent stage in serum of infected pigs (Figures 2B,C), indicating less severe inflammation by SDP.

Porcine rotavirus targets enterocytes to induce acute viral gastroenteritis. The jejunum is the major effector site for viral infection, and the activation of mucosal immunity is critical for pathogen clearance and tissue repair. RV infection only induced mucosal IL-1β (*p* = 0.041) production during the manifestation stage, and increased most of mucosal inflammatory cytokines, such as mucosal TNF-α (*p* = 0.045), IL-10 (*p* = 0.026), IL-1β (*p* = 0.036), and TGFβ (*p* = 0.019) until 6 days post infection, whose pattern was different from RV-induced systemic inflammation (Figures 2E,F). Notably, in RV-infected pigs, SDP in the diet significantly increased IFN-γ (*p* = 0.015), TNF-α (*p* = 0.034), IL-6 (*p* = 0.057), and IL-10 (*p* = 0.041) in jejunal mucosa during the manifestation stage, while SDP decreased most of inflammatory cytokines during the convalescent stage (Figures 2E,F), in which SDP exhibited the opposite action against RV infection. The mRNA expressions of cytokines and NFκB signaling related genes were investigated in jejunal mucosa, and SDP acted against RV-induced changes on *IL-6*, *MCP1*, *TNF-α*, *TGFβ* and *MyD88* expressions (Supplementary Figure S1).

These results indicated that SDP promoted mucosal immunity upon RV infection but alleviated RV-induced systemic inflammation, as well as maintaining the immune homeostasis.

### Spray-dried bovine plasma modulates immunoglobulins and RV-ab in weanling pigs

**Figure 3:**
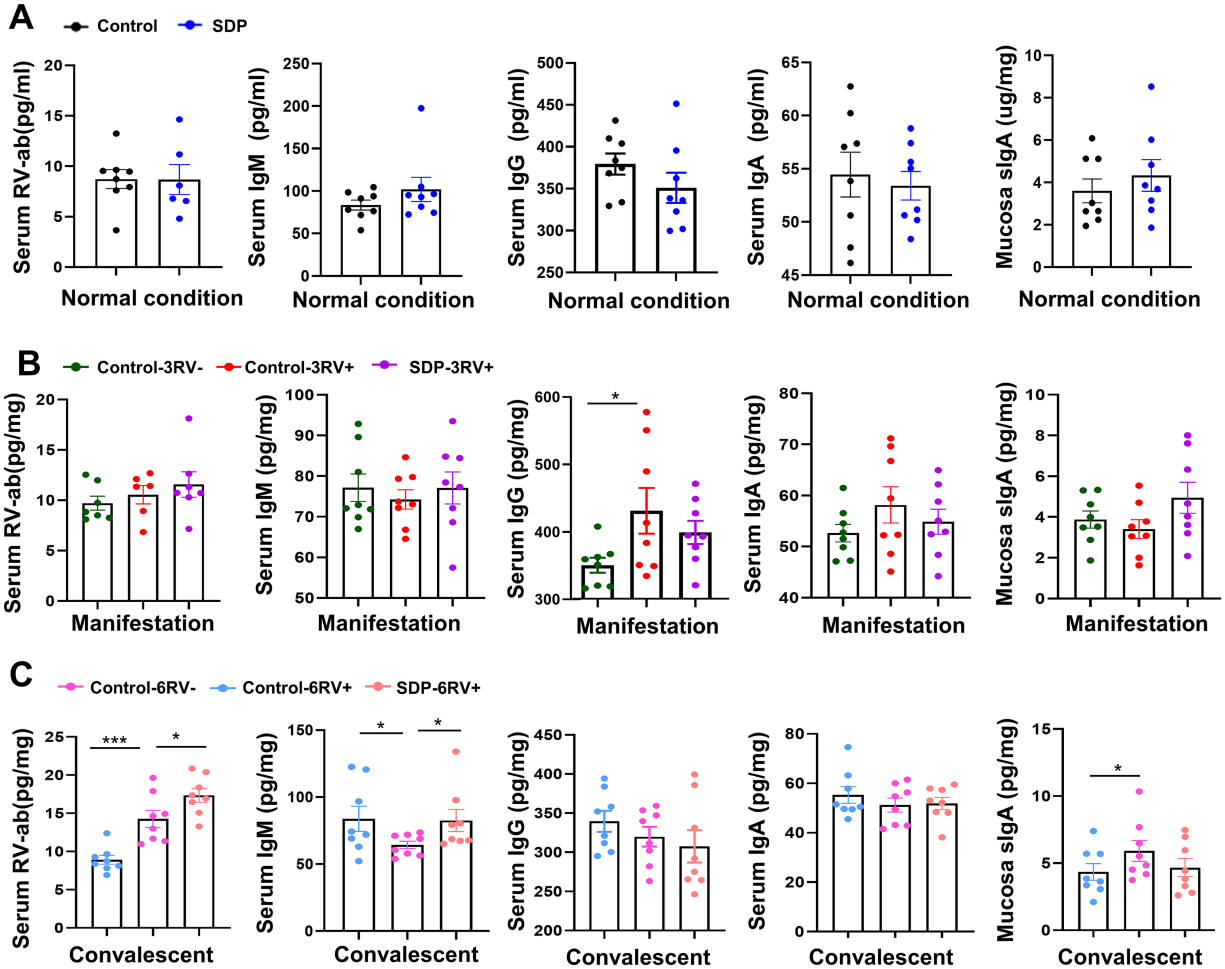
Spray-dried bovine plasma modulates immunoglobulins and RV-ab in weanling pigs. **(A)** Under normal condition, levels of RV-ab, IgM, IgG and IgA in serum and sIgA in ileal mucosa in control and SDP groups. Data are expressed as mean ± SEM (*n* = 8). **(B)** During the manifestation stage, levels of RV-ab, IgM, IgG and IgA in serum and sIgA in ileal mucosa of control-3RV−, control-3RV+ and SDP-3RV+ groups. Data are expressed as mean ± SEM (*n* = 8). **(C)** During the convalescent stage, levels of RV-ab, IgM, IgG and IgA in serum and sIgA in ileal mucosa of control-6RV−, control-6RV+ and SDP-6RV+ groups. Data are expressed as mean ± SEM (*n* = 8).

The levels of immunoglobulin and RV-antibody were measured in serum and jejunum mucosa. Under normal conditions, RV-antibody (RV-ab), IgM, IgG and IgA levels in serum, and sIgA concentration in jejunal mucosa were not influenced by SDP supplementation (Figure 3A). RV challenge gradually increased RV-ab (*p* = 0.0008) level and reached significance during the convalescent stage, while SDP further increased RV-ab (*p* = 0.049) in RV-infected pigs, indicating the enhancement on immunity by SDP supplementation. During the manifestation stage, RV infection increased serum IgG (*p* = 0.039) and numerically increased IgA, while SDP in the diet maintained serum immunoglobulin levels close to unchallenged pigs; of note, SDP numerically increased mucosal sIgA concentration in RV-infected pigs (Figure 3B). During convalescent, RV decreased serum IgM (*p* = 0.022) and increased mucosal sIgA (*p* = 0.041), and similarly SDP acted against RV-induced changes (Figure 3C). These results indicated that SDP enhanced immunity upon RV-infection and maintained immune homeostasis like that of uninfected pigs.

### Spray-dried bovine plasma alters immune cell population in weanling pigs

**Figure 4:**
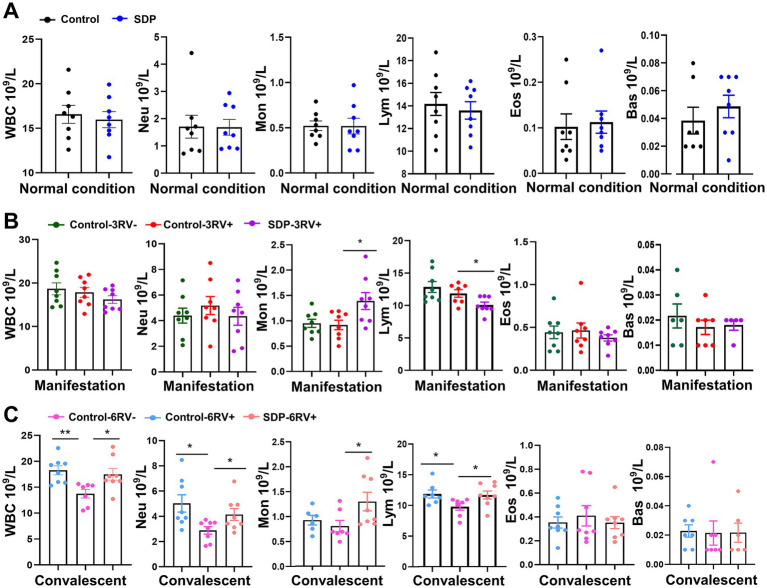
Spray-dried bovine plasma alters immune cell population in weanling pigs. **(A)** Under normal condition, the number of WBC, Neu, Mon, Lym, Eos and Bas cells contained in the blood of control and SDP groups. Data are expressed as mean ± SEM (*n* = 8). **(B)** During the manifestation stage, the number of WBC, Neu, Mon, Lym, Eos and Bas cells contained in the blood of control-3RV−, control-3RV+ and SDP-3RV+ groups. Data are expressed as mean ± SEM (*n* = 8). **(C)** During the convalescent stage, the number of WBC, Neu, Mon, Lym, Eos and Bas cells contained in the blood of control-6RV−, control-6RV+ and SDP-6RV+ groups. Data are expressed as mean ± SEM (*n* = 8).

**Figure 5:**
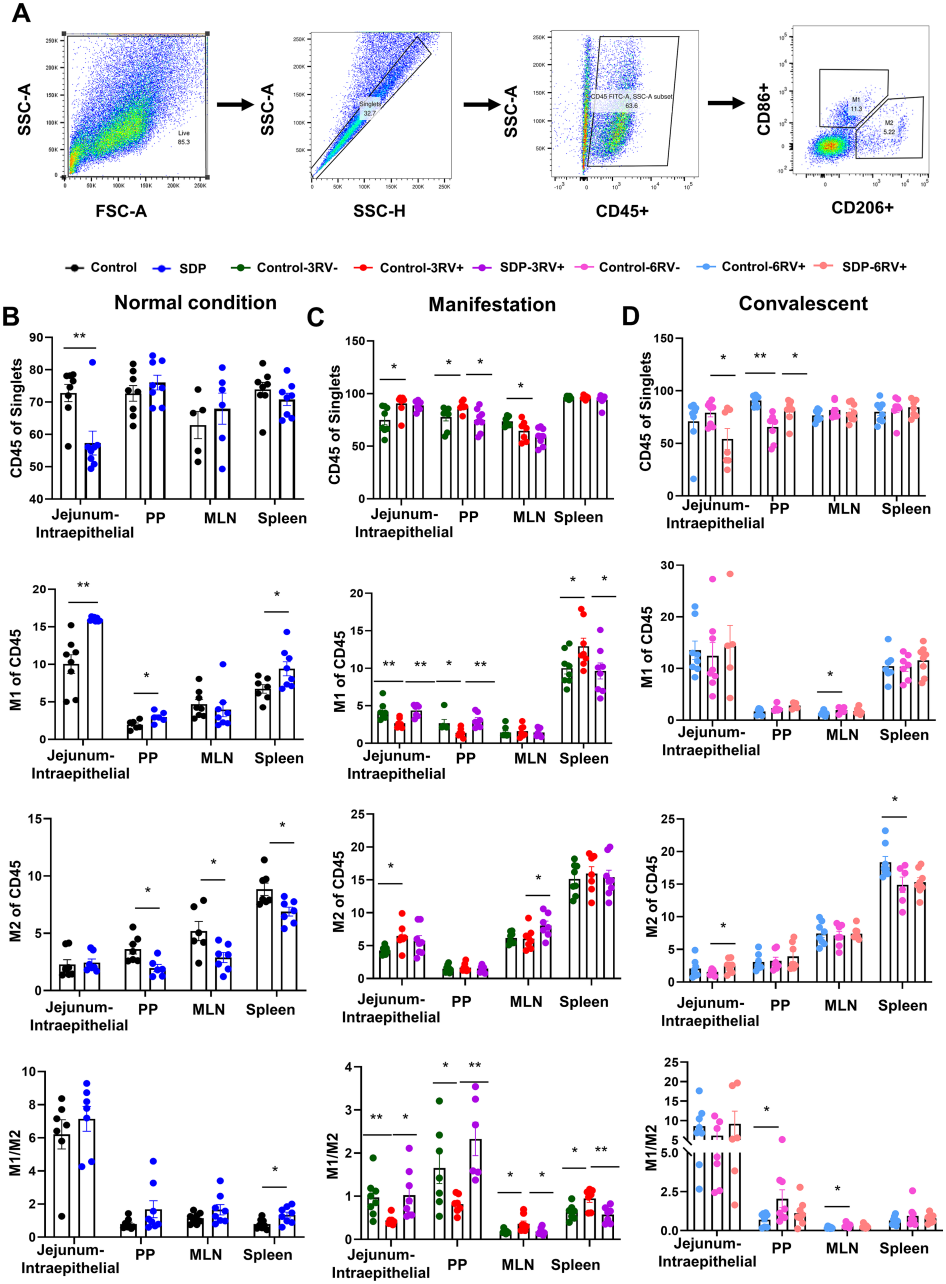
Spray-dried bovine plasma modulates macrophage polarization in weanling pigs upon RV challenge. **(A)** Schematic diagram of gates for immune cells and M1 and M2 macrophages in flow cytometry experiments. **(B)** Under normal condition, the change of immune cells (CD45^+^), M1 macrophages (CD86^+^), M2 macrophages (CD206^+^) and the ratio of M1/M2 in jejunum-intraepithelial, PP, MLN and spleen tissues of control and SDP groups. Data are expressed as mean ± SEM (*n* = 8). **(C)** During the manifestation stage, the change of immune cells (CD45^+^), M1 macrophages (CD86^+^), M2 macrophages (CD206^+^) and the ratio of M1/M2 in jejunum-intraepithelial, PP, MLN and spleen tissues of control-3RV−, control-3RV+ and SDP-3RV+ groups. Data are expressed as mean ± SEM (*n* = 8). **(D)** During the convalescent stage, the change of immune cells (CD45^+^), M1 macrophages (CD86^+^), M2 macrophages (CD206^+^) and the ratio of M1/M2 in jejunum-intraepithelial, PP, MLN and spleen tissues of control-6RV−, control-6RV+ and SDP-6RV+ groups. Data are expressed as mean ± SEM (*n* = 8).

The immune cell population was analyzed in fresh blood samples. SDP in the diet had no significant effects on the basal immune cell population prior to RV infection (Figure 4A). RV infection slightly influenced immune cell populations during the manifestation stage, while RV-infected pigs fed SDP exhibited increased monocytes (*p* = 0.026) and decreased lymphocytes (*p* = 0.028) during this stage (Figure 4B), indicating the rapid activation of immune response by SDP upon RV infection. During convalescent, RV significantly decreased total white blood cells (WBC) (*p* = 0.002), neutrophils (*p* = 0.012), and lymphocytes (*p* = 0.032), while SDP showed the opposite effect against RV infection (Figure 4C). We next determined the changes in immune cell (CD45^+^ positive cell) population in the single cell suspension from jejunum intraepithelial cells, PP, MLN and spleen. RV infection significantly increased immune cells in jejunum intraepithelial (*p* = 0.012) and PP (*p* = 0.038), and decreased cells in MLN (*p* = 0.044) during manifestation, and decreased immune cells in PP (*p* = 0.0001) during convalescent (Figure 5B). In RV-infected pigs, SDP in the diet also acted against the RV-induced changes of CD45^+^ cell population in jejunum intraepithelial (*p* = 0.029) and PP (*p* = 0.012) (Figure 5B).

### Spray-dried bovine plasma modulates macrophage polarization in weanling pigs upon RV challenge

Macrophage polarization was analyzed in CD45^+^ cells of single cell suspension from different tissues as mentioned above; CD206 and CD86 were used to identify M2 and M1 macrophages, respectively (Figure 5A). Prior to RV challenge, SDP in diet significantly increased M1 population in PP (*p* = 0.021), jejunal intraepithelial (*p* = 0.0003) and spleen (*p* = 0.033), and decreased M2 population in PP (*p* = 0.011), MLN (*p* = 0.026), and spleen (*p* = 0.013), and increased the M1/M2 ratio in all these tissues (Figure 5B), indicating the enhancement on basal innate immunity by SDP. During manifestation, RV-infection significantly decreased M1, increased M2 (*p* = 0.014) population and decreased the M1/M2 ratio in PP and jejunal intraepithelial (Figure 5C); on the contrary, RV increased M1 (*p* = 0.042) percentage and the M1/M2 (*p* = 0.017, *p* = 0.015) ratio in MLN and spleen (Figure 5C), indicating the disparity in macrophage population in the intestine and immune organs. Notably, in RV-infected pigs, SDP in diet totally reversed the RV-induced changes of M1population and M1/M2 ratio in all four tissues (Figure 5C). Additionally, SDP also significantly increased M2 population in MLN (*p* = 0.040). During convalescent, RV infection led to increases in the M1 (*p* = 0.048) population in MLN and the M1/M2 ratio in PP and MLN, and the decrease in M2 (*p* = 0.034) population in intraepithelial and spleen, in RV-infected pigs, SDP increased M2 (*p* = 0.036) population in intraepithelial, decreased the M1/M2 ratio in PP and MLN, and recovered macrophage population close to normal condition in different tissues (Figure 5D).

These results showed that SDP in diet increased basal M1 population, guaranteed the sufficient M1 macrophage involved in innate immunity during the manifestation stage, and facilitated immunity homeostasis and tissue repair during the convalescent stage. RV-infected pigs without SDP exhibited delayed immune response.

### Spray-dried bovine plasma regulates T lymphocyte divergence in weanling pigs upon RV challenge

**Figure 6:**
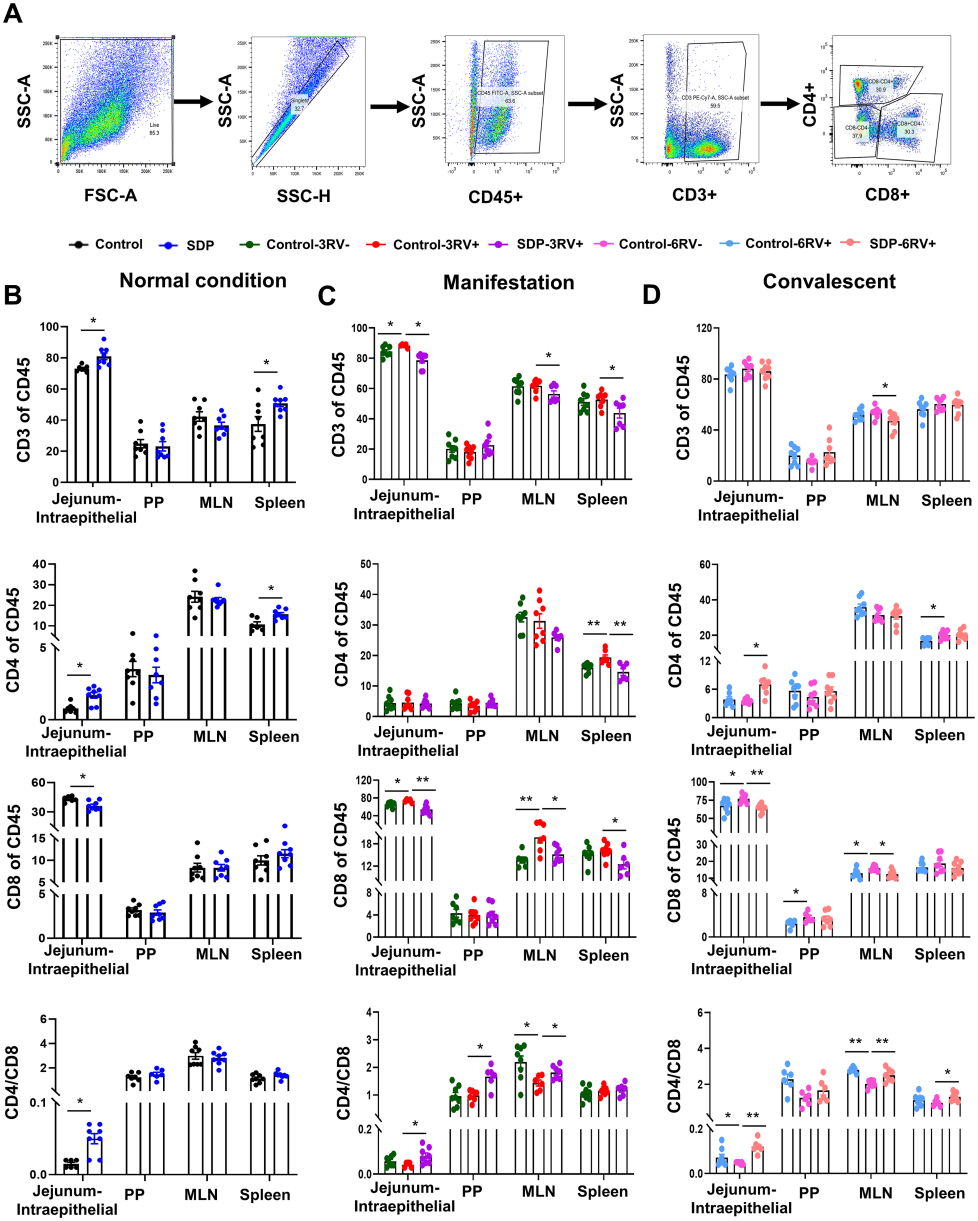
Spray-dried bovine plasma regulates T lymphocyte divergence in weanling pigs upon RV challenge. **(A)** Schematic diagram of gates for lymphocytes, helper T lymphocytes and cytotoxic T lymphocytes in flow cytometry experiments. **(B)** Under normal condition, the change of immune cells (CD45^+^), lymphocytes (CD3^+^), helper T lymphocytes (CD4^+^), cytotoxic T lymphocytes (CD8^+^) and CD4^+^/CD8^+^ ratio in jejunum-intraepithelial, PP, MLN and spleen tissues of control and SDP groups. Data are expressed as mean ± SEM (*n* = 8). **(C)** During the manifestation stage, the change of immune cells (CD45^+^), lymphocytes (CD3^+^), helper T lymphocytes (CD4^+^), cytotoxic T lymphocytes (CD8^+^) and CD4^+^/CD8^+^ ratio in jejunum-intraepithelial, PP, MLN and spleen tissues of control-3RV−, control-3RV+ and SDP-3RV+ groups. Data are expressed as mean ± SEM (*n* = 8). **(D)** During the convalescent stage, the change of immune cells (CD45^+^), lymphocytes (CD3^+^), helper T lymphocytes (CD4^+^), cytotoxic T lymphocytes (CD8^+^) and CD4^+^/CD8^+^ ratio in jejunum-intraepithelial, PP, MLN and spleen tissues of control-6RV−, control-6RV+ and SDP-6RV+ groups. Data are expressed as mean ± SEM (*n* = 8).

T lymphocyte divergence was further analyzed in CD45^+^ cells of single cell suspension from different tissues as mentioned above. CD3 cells were used as a marker of total T lymphocytes, and subsequently CD4 and CD8 were used to identify regulatory T cells (Treg) and cytotoxic T cells, respectively (Figure 6A). Prior to RV challenge, SDP in diet significantly increased all lymphocytes, CD4^+^ cells, and the CD4^+^/CD8^+^ (*p* = 0.003) ratio in jejunal intraepithelial and spleen, and decreased CD8^+^ (*p* = 0.029) population in jejunal intraepithelial (Figure 6B), indicating less immune senescence and chronic inflammation in normal conditions. During manifestation, RV-infection exhibited a slight influence on T cell divergence in that RV only significantly increased CD8 cells (*p* = 0.004) in jejunal intraepithelial and MLN (*p* < 0.05) and decreased the CD4^+^/CD8^+^ (*p* = 0.042) ratio in MLN (Figure 6C). In RV-infected pigs, SDP in diet significantly (*p* < 0.05) decreased CD3^+^ and CD8^+^ cells in jejunal intraepithelial, MLN and spleen, and increased CD4^+^/CD8^+^ ratio in jejunal intraepithelial, PP and MLN (*p* < 0.05; Figure 6C), suggesting a robust influence on lymphocytes population. During convalescent, RV infection led to increases (*p* < 0.05) in CD8^+^ cell population in intraepithelial, PP and MLN, and a reduction of CD4^+^/CD8^+^ in jejunal intraepithelial (*p* < 0.05) and MLN (*p* = 0.001, Figure 6D), In RV-infected pigs, SDP decreased CD3^+^ (*p* = 0.022) in MLN and CD8^+^ (*p* < 0.0001) cells in intraepithelial (*p* < 0.001) and MLN (*p* < 0.05), and increased CD4^+^ (*p* = 0.001) cells in intraepithelial (*p* < 0.05) and CD4^+^/CD8^+^ ratio in intraepithelial (*p* < 0.001) and MLN (*p* < 0.001).

These results showed that effects of RV infection and SDP supplementation had no disparity among different stages, respectively. Pigs fed SDP exhibited an increase in CD8^+^ cells, and a decrease CD4^+^ cells and CD4^+^/CD8^+^ ratio, indicating a more robust immune function with less chronic inflammation.

## Discussion

In infants and young animals, the intestines are still in developing, with gastrointestinal function and immune systems not yet fully established. This leads to an incomplete intestinal barrier, making them highly vulnerable to exogenous pathogens. As a result, they are at greater risk of intestinal damage, nutrient malabsorption, diarrhea, and even mortality (22–25). In this study, weanling pigs suffered from severe diarrhea in the first week of the trial, resulting from weaning stress, long-distance transportation and environmental changes. SDP supplementation alleviated stresses as shown by the reduced incidence of diarrhea and the enhanced feed conversion efficiency. A positive correlation of nutrient digestibility to villus height and villus-to-crypt ratio has been established (26–28). SDP improved intestinal morphology and increased tight junction expression, thereby contributing to the enhanced growth performance. Furthermore, studies have demonstrated that SDP alleviates stresses and improves intestinal barriers function by enhancing anti-oxidative capacity (29–31).

Rotavirus primarily lodges inside the enterocytes to induce osmotic diarrhea and disrupts the cytoskeleton and tight junctions to impair barrier integrity (32). Rotavirus secretes NSP4 acting as a viral enterotoxin to trigger electrolyte imbalance and secretory diarrhea (5, 33). Our study revealed the RV-induced disruption of intestinal morphology and tight junctions resulted in reduced growth performance and severe diarrhea. We demonstrated the disease resistance properties of SDP from multiple perspectives, including diarrhea incidence, growth performance, intestinal health (such as intestinal morphology, tight junction integrity, and epithelium apoptosis), as well as immune indicators (including immune molecules and immune cell populations). SDP in diet exhibited the protective benefit against RV-induced acute viral gastroenteritis in weanling pigs, but the underlying mechanism was rarely elucidated. Studies shown that SDP reduced the virus-binding protein in the intestines of RV-infected piglets and resists viral invasion (12). Research indicated that SDP mitigated the viral shedding of PEDV and stimulated an earlier PEDV antibody response (16), and the increased RV antibody production were also observed in our study. In COVID-19 patients, convalescent plasma therapy reduced hospitalization period, disease severity and mortality, but also reduced the recurrence rate (34, 35). These evidences indicated that plasma acts as an immune modulator against viral infection; however, there is no research focused on the regulation of the immune response by SDP during the progression of RV infection.

Innate immunity acts as the first line of defense against pathogens in mammals, with macrophages playing a central role. M1 macrophages, or classically activated macrophages, are responsible for initiating pro-inflammatory responses, recognizing pathogens, and producing cytokines to eliminate infections (36). On the other hand, M2 macrophages, also known as alternatively activated macrophages, focus on anti-inflammatory responses, tissue repair, and restoring immune balance after infections (35, 37–39). The dynamic balance between M1’s pathogen-fighting role and M2’s healing and recovery function is essential for maintaining overall immune health in animals (40). Our study revealed that under normal conditions, SDP increased the number of M1 macrophages in different immune tissues, accounting for the rapid immune response in SDP-fed pigs. Notably, the M1/M2 ratio in SDP was at a normal level, and the levels of inflammatory factors in the blood and ileal mucosal tissues were not affected, indicating that SDP increased the body’s sensitivity to pathogens under normal conditions and did not induce inflammation. Notably, M1/M2 ratio in the SDP group remained unchanged, and the levels of inflammatory factors in both the serum and ileal mucosa were unaffected, suggesting that SDP enhanced immune sensitivity to pathogens under normal conditions, without inducing inflammation.

During the manifestation stage of RV infection, SDP enhanced the release of inflammatory cytokines in the mucosa but inhibited the release of inflammatory cytokines in the serum. SDP increased M1 number and M1/M2 ratio in the intestinal interstitial and PP tissues, promoted mucosal immune response, and reduced the number of M1 cells and M1/M2 ratio in peripheral immune tissues. These results are consistent, indicating that changes in blood and mucosal cytokines can be explained by variations in M1, M2, and the M1/M2 ratio across different tissues. SDP improved the mucosal immunity and prevented the spread of the rotavirus but did not affect systemic immunity. During the convalescent stage of RV infection, RV elevated mucosal inflammatory cytokines, promoted macrophage differentiation into M1 cells in immune tissues, increased the M1/M2 ratio, and contributed to prolonged inflammation. In contrast, SDP promoted M2 differentiation and inhibited M1 polarization, thereby reducing chronic inflammation and supporting tissue recovery. The findings indicated that SDP modulates macrophage population differentiation across different tissues, thereby altering systemic and mucosal immune balance to regulate systemic inflammation, pathogen recognition and clearance, as well as tissue repair.

Lymphocytes are critical for pathogen recognition and clearance, as well as inflammation regulation (18). Viral infections, such as ASFV and the RV infection involved in our study, result in a decline in white blood cells, primarily lymphocytes (41). In severe cases, this can lead to lymphopenia, significantly impairing the immune response and hindering pathogen clearance (42). SDP increased the number of white blood cells and lymphocytes in the blood to resist viral invasion. CD4^+^ helper T cells are pivotal in coordinating both humoral and cell-mediated immune responses, and a decrease in CD4^+^ T cell numbers indicates immune insufficiency (43). CD8^+^ cytotoxic T cells are vital for viral clearance by directly killing infected cells, but uncontrolled CD8^+^ T cells lead to collateral damage to healthy cells (43). Under normal conditions, the SDP diet increased the number of CD4^+^ T lymphocytes and decreased the number of CD8^+^ T lymphocytes, indicating the enhancement of immunity as previously mentioned. Upon RV challenge, piglets supplemented with SDP showed a decrease in CD8^+^ cells, an increase in CD4^+^ T cells, and a higher CD4^+^/CD8^+^ ratio. Studies have shown that persistently low CD4^+^/CD8^+^ ratios during virus infection are associated with immunosenescence and elevated risks of morbidity and mortality (44, 45). Therefore, our results demonstrate that SDP can enhance the immune functions in piglets. Moreover, research on acute SARS-CoV-2 infections found that patients with lower CD8^+^ levels and higher CD4^+^/CD8^+^ ratios did not present more severe acute infection or inflammation (46). Considering the earlier discussion that SDP accelerates immune responses and pathogen clearance, along with the potential adverse effects of excessive CD8^+^ cells, the reduction in CD8^+^ cells induced by SDP may indicate an opportunity for quicker recovery and a return to immune homeostasis.

In addition to our study, SDP was reported to alleviate intestinal damage caused by porcine epidemic diarrhea virus (PEDV). Research also demonstrated that SDP mitigated infections from non-enteric diseases, such as porcine reproductive and respiratory syndrome (PRRS) and African swine fever (ASF). Evidence also suggested that SDP enhances overall health in poultry and ruminants (47, 48). These findings indicated that the antiviral properties of SDP may extend beyond pigs and rotavirus, suggesting its broader potential application in combating a wide range of diseases and infections across multiple livestock species.

## Conclusion

Under normal conditions, SDP increases the baseline number of immune cells in piglets without activating an immune response. During the outbreak phase of RV infection, SDP promotes a more rapid immune response, characterized by enhanced mucosal immunity without triggering systemic inflammation. In the recovery phase, SDP supports the replenishment of immune cells, helping to restore immune homeostasis, and facilitates anti-inflammatory processes and tissue repair. We have explained the immunomodulatory mechanisms of SDP at the macrophage and T cell levels. Additionally, SDP has demonstrated potential in combating non-RV viruses, such as PRRSV, PEDV, and ASFV, and shows promise in modulating immunity and enhancing disease resistance across different species, indicating significant potential for broader applications.

## Data Availability

The datasets presented in this study can be found in online repositories. The names of the repository/repositories and accession number(s) can be found in the article/Supplementary material.

## Supplementary material

SUPPLEMENTARY FIGURE S1 The mRNA expression of inflammation related markers in jejunal mucosa. Spray-dried bovine plasma modulates immunoglobulins and RV-ab in weanling pigs. (A) Under normal condition, the expression levels of inflammatory cytokine-related genes in jejunal mucosa of control and SDP groups. Data are expressed as mean ± SEM (*n* = 8). (B) During the manifestation stage, the expression levels of inflammatory cytokine-related genes in jejunal mucosa of control-3RV−, control-3RV+ and SDP-3RV+ groups. Data are expressed as mean ± SEM (*n* = 8). (C) During the convalescent stage, the expression levels of inflammatory cytokine-related genes in jejunal mucosa of control-6RV−, control-6RV+ and SDP-6RV+ groups. Data are expressed as mean ± SEM (*n* = 8).
