# Supplementary figures and images for: Spray-dried plasma protects against rotavirus-induced gastroenteritis via regulating macrophage and T cells divergence in weanling pigs
Source: Front Vet Sci. 2024 Oct 16;11:1467108. doi: 10.3389/fvets.2024.1467108 (PMC11523297; doi:10.3389/fvets.2024.1467108)

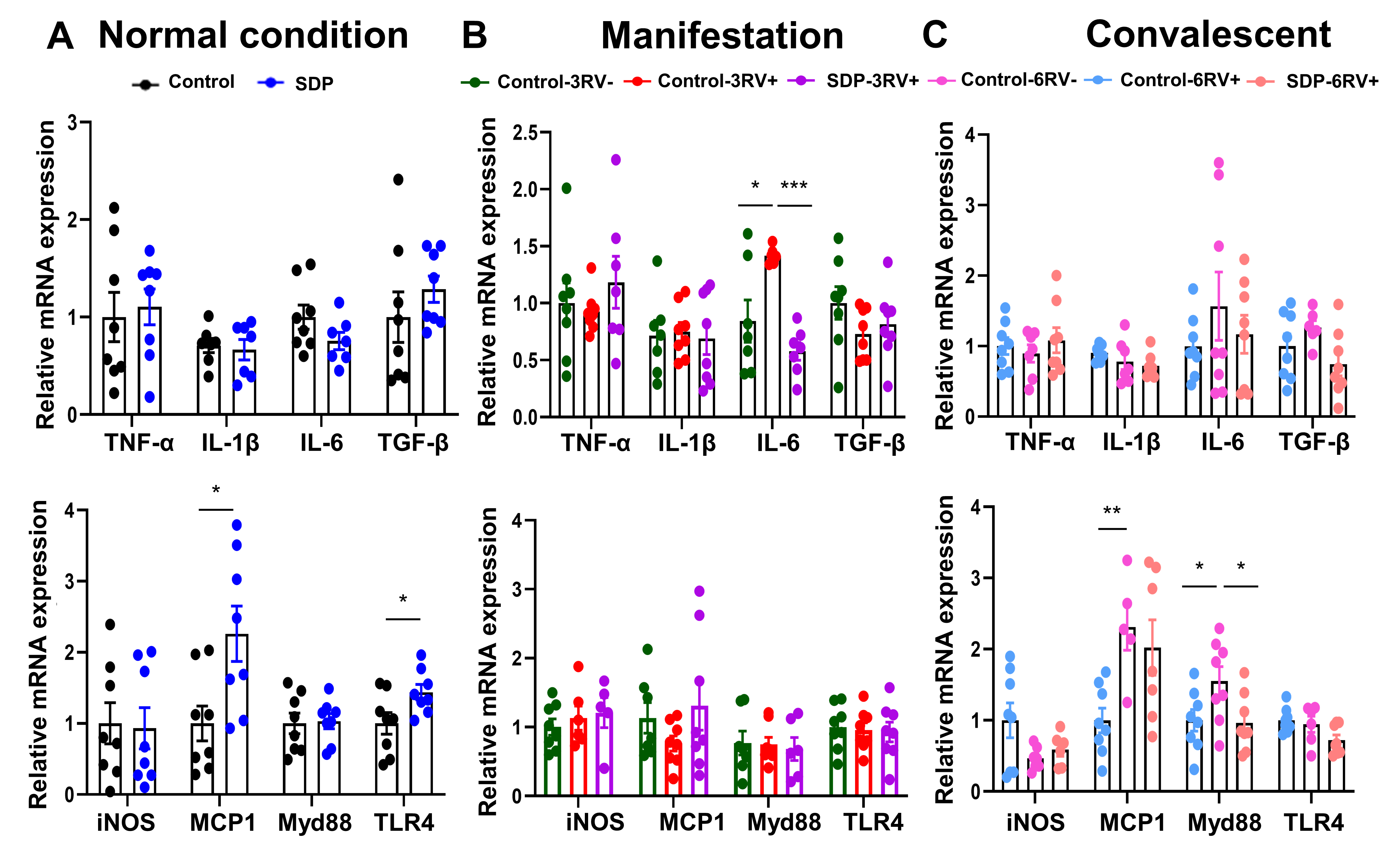

Supplement: SUPPLEMENTARY FIGURE S1 — The mRNA expression of inflammation related markers in jejunal mucosa. Spray-dried bovine plasma modulates immunoglobulins and RV-ab in weanling pigs. (A) Under normal condition, the expression levels of inflammatory cytokine-related genes in jejunal mucosa of control and SDP groups. Data are expressed as mean ± SEM (n = 8). (B) During the manifestation stage, the expression levels of inflammatory cytokine-related genes in jejunal mucosa of control-3RV−, control-3RV+ and SDP-3RV+ groups. Data are expressed as mean ± SEM (n = 8). (C) During the convalescent stage, the expression levels of inflammatory cytokine-related genes in jejunal mucosa of control-6RV−, control-6RV+ and SDP-6RV+ groups. Data are expressed as mean ± SEM (n = 8). [file Image_1.tif]
