# Supplementary material for: Spray-dried plasma protects against rotavirus-induced gastroenteritis via regulating macrophage and T cells divergence in weanling pigs
Source: Front Vet Sci. 2024 Oct 16;11:1467108. doi: 10.3389/fvets.2024.1467108 (PMC11523297; doi:10.3389/fvets.2024.1467108)
Supplement: Supplementary file 2 [file Table_1.docx]

| **TableS1.** Composition and nutrient concentrations of experimental diets | | |
| --- | --- | --- |
| Ingredients | Formula（%） | |
|  | Control | 6% SDP |
| Corn, 7.8% CP | 59.56 | 61.69 |
| Soybean Meal, 43%CP | 16.5 | 16.5 |
| Fish Meal, 62.5% CP | 3 | 3 |
| Whey Premeate, 3.8% CP | 5 | 5 |
| SPC, 65.2% CP | 6.7 | 0 |
| Soy Oil | 3.6 | 3 |
| Sugar | 0.5 | 0.5 |
| Limstone | 0.41 | 0.7 |
| Monocalcium Phosphate | 1.4 | 1 |
| L-lysine-HCl, 78% | 0.56 | 0.38 |
| DL Methionine, 99% | 0.22 | 0.24 |
| L-Threonine, 98.5% | 0.2 | 0.1 |
| Trp, 98% | 0.07 | 0.03 |
| Valine, 98.5% | 0.14 | 0.02 |
| SDBP, 78% CP | 0 | 6 |
| NaCl | 0.65 | 0.35 |
| Trace Mineral Premix^1^ | 0.2 | 0.2 |
| Vitamin Premix^2^ | 0.2 | 0.2 |
| Choline Chloride, 50% | 0.1 | 0.1 |
| Acidifiers | 1 | 1 |
| Total | 100 | 100 |
| **Calculated Nutrition Levels^3^** | | |
| DE (kcal/kg) | 3466.16 | 3446.93 |
| ME (kcal/kg) | 3308.86 | 3308.22 |
| CP | 19.25 | 19.35 |
| Ca | 0.8 | 0.79 |
| Total P | 0.7 | 0.66 |
| Av.P (ATTD P) | 0.43e | 0.43 |
| SID-Lys | 1.35 | 1.35 |
| SID-Met | 0.49 | 0.49 |
| SID-Thr | 0.81 | 0.81 |
| SID-Trp | 0.25 | 0.25 |
| SID-Met + Cys | 0.74 | 0.83 |
| CP = crude protein; Ash = crude ash; CF = crude fiber; EE = ether extract; GE = gross energy; DE = digestible energy; ME = metabolizable energy.  ^1^The vitamin premix provided the following per kg of diet: 6000 IU of VA, 3000 IU of VD 3, 24 IU of VE, 3 mg of VK3, 1.5 mg of VB1, 6 mg of VB 2, 3 mg of VB6, 0.02 mg of VB12, 14 mg of niacin, 15 mg of pantothenic acid, 0.75 mg of folic acid, and 0.1 mg of biotin.  ^2^The mineral premix provided the following per kg of diet: Fe （FeSO4·H2O）, 100 mg; Cu （CuSO4·5H2O）, 6 mg; Mn （MnSO4·H2O）, 4 mg; Zn （ZnSO4·H2O）, 100 mg; I （KI）, 0.3 mg; Se （Na2SeO3）, 0.3 mg.  ^3^Nutrient level values were calculated | | |
