# Supplementary material for: Spray-dried plasma protects against rotavirus-induced gastroenteritis via regulating macrophage and T cells divergence in weanling pigs
Source: Front Vet Sci. 2024 Oct 16;11:1467108. doi: 10.3389/fvets.2024.1467108 (PMC11523297; doi:10.3389/fvets.2024.1467108)
Supplement: Supplementary file 3 [file Table_2.docx]

| **Table S2.** Primer sequences for quantitative real-time PCR | | | |
| --- | --- | --- | --- |
| Primer names |  | Nucleotide sequence (5′–3′) |  |
| β-actin | Forward | TCTGGCACCACACCTTCT |  |
|  | Reverse | TGATCTGGGTCATCTTCTCAC |  |
| ZO-1 | Forward | CAGCCCCCGTACATGGAGA |  |
|  | Reverse | GCGCAGACGGTGTTCATAGTT |  |
| Claudin-1 | Forward | TCTTAGTTGCCACAGCATGG |  |
|  | Reverse | CCAGTGAAGAGAGCCTGACC |  |
| Occludin | Forward | CTACTCGTCCAACGGGAAAG |  |
|  | Reverse | ACGCCTCCAAGTTACCACTG |  |
| MUC1 | Forward | GTGCCGCTGCCCACAACCTG |  |
|  | Reverse | AGCCGGGTACCCCAGACCCA |  |
| MUC2 | Forward | GGTCATGCTGGAGCTGGACAGT |  |
|  | Reverse | TGCCTCCTCGGGGTCGTCAC |  |
| Bax | Forward | AAGCGCATTGGAGATGAACT |  |
|  | Reverse | TGCCGTCAGCAAACATTTC |  |
| Bcl | Forward | GCTACTTACTGCCAAAGGGA |  |
|  | Reverse | TTCAGGCGGAGCTGTAAGAG |  |
| Bad | Forward | GAGTCGCCACAGCTCTTACC |  |
|  | Reverse | GCGAGGAAGTCCCTTCTTGA |  |
| TNF-α | Forward | CGTGAAGCTGAAAGACAACCAG |  |
|  | Reverse | GATGGTGTGAGTGAGGAAAACG |  |
| IL-1β | Forward | AGCCCATCGTCCTTGAAAAGC |  |
|  | Reverse | GCACAAAGCTCATGCAGAACA |  |
| IL-6 | Forward | TTCACCTCTCCGGACAAAAC |  |
|  | Reverse | TCTGCCAGTACCTCCTTGCT |  |
| TGF-β | Forward | GCCGGAACCTGTATTGCTCT |  |
|  | Reverse | CTCTATAGCCTCTCTGCGGGTCA |  |
| iNOS | Forward | GAGCCCAGAGGGCTTTATCA |  |
|  | Reverse | TTCTTTGCTGTCTCCGCCAG |  |
| MCP1 | Forward | ATTCTCCAGTCACCTGCTGC |  |
|  | Reverse | TGCTGGTGACTCTTCTGTAGC |  |
| Myd88 | Forward | GTGCCGTCGGATGGTAGTG |  |
|  | Reverse | TCTGGAAGTCACATTCCTTGCTT |  |
| TLR4 | Forward | TATTGTCGTGGTGTCCCAGC |  |
|  | Reverse | TGTCCTCCCACTCCAGGTAG |  |
